# Flexible Magnetocaloric Fiber Mats for Room-Temperature Energy Applications

**DOI:** 10.1021/acsami.3c15833

**Published:** 2024-02-01

**Authors:** Vahideh Bayzi Isfahani, Indrani Coondoo, Igor Bdikin, Konstantin Skokov, João Ricardo da Silva Gomes, Rosa Maria Ferreira Baptista, Clara Rodrigues Pereira, João Pedro Araújo, Michael Scott Belsley, Etelvina de Matos Gomes, João Horta Belo, Bernardo Gonçalves Almeida

**Affiliations:** † Centre of Physics of Minho and Porto Universities (CF-UM-UP), LAPMET, Physics Department, University of Minho, Campus of Gualtar, 4710-057 Braga, Portugal; ‡ Department of Physics & CICECO − Aveiro Institute of Materials, University of Aveiro, 3810-193 Aveiro, Portugal; § TEMA: Centre for Mechanical Technology and Automation, Department of Mechanical Engineering, University of Aveiro, Campus de Santiago, 3810-193 Aveiro, Portugal; ∥ LASI−Intelligent Systems Associate Laboratory, 4800-058 Guimarães, Portugal; ⊥ Institute of Materials Science, Technical University of Darmstadt, Alarich-Weiss-Straße 16, 64287 Darmstadt, Germany; # REQUIMTE/LAQV, Department of Chemistry and Biochemistry, Faculty of Sciences, University of Porto, 4169-007 Porto, Portugal; ∇ Institute of Physics of Advanced Materials, Nanotechnology and Photonics (IFIMUP), Department of Physics and Astronomy, Faculty of Sciences, University of Porto, Rua Campo Alegre, 4169-007 Porto, Portugal

**Keywords:** multifunctional and flexible fibers, PVDF polymer matrix, La(FeSi)_13_ powder, electrospinning, piezoelectric, magnetocaloric, energy applications

## Abstract

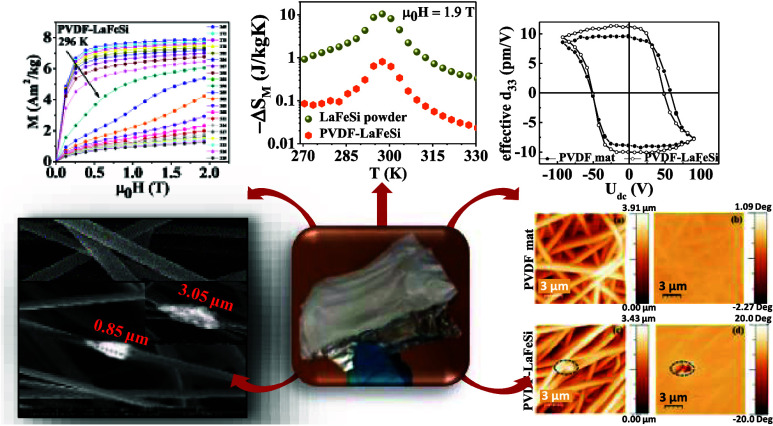

Currently, magnetocaloric refrigeration technologies are emerging as ecofriendly and more energy-efficient alternatives to conventional expansion–compression systems. However, major challenges remain. A particular concern is the mechanical properties of magnetocaloric materials, namely, their fatigue under cycling and difficulty in processing and shaping. Nevertheless, in the past few years, using multistimuli thermodynamic cycles with multicaloric refrigerants has led to higher heat-pumping efficiencies. To address simultaneously the challenges and develop a multicaloric material, in this work, we have prepared magnetocaloric-based flexible composite mats composed of micrometric electroactive (EA) polyvinylidene fluoride (PVDF) fibers with embedded magnetocaloric/strictive La(Fe,Si)_13_ particles by the simple and cost-effective electrospinning technique. The composite’s structural characterization, using X-ray diffraction (XRD) analysis, Fourier transform infrared (FTIR) spectroscopy, and measurements of the local-scale piezoresponse, revealed a cubic NaZn_13_-type structure of the La(Fe,Si)_13_ phase and the formation of the dominant polar β-phase of the PVDF polymer. The PVDF-La(Fe,Si)_13_ composite showed an enhancement of the longitudinal piezoelectric coefficient (effective d_33_) (−11.01 pm/V) compared with the single PVDF fiber matrix (−9.36 pm/V). The main magnetic properties of La(Fe,Si)_13_ powder were retained in the PVDF-La(Fe,Si)_13_ composite, including its giant magnetocaloric effect. By retaining the unique magnetic properties of La(Fe,Si)_13_ embedded in the electroactive piezoelectric polymer fiber mats, we have designed a flexible, easily shapeable, and multifunctional composite enabling its potential application in multicaloric heat-pumping devices and other sensing and actuating devices.

## 1 Introduction

The magnetocaloric effect (MCE) serves as the basis for two promising energy related technologies: magnetic refrigeration and thermomagnetic waste heat energy harvesting. It manifests as an adiabatic temperature change (Δ*T*_ad_) or an isothermal entropy change (Δ*S*_m_) in a magnetic material when a magnetic field is applied.^1−3^ Similar to gas compression cycles used in current cooling technologies, the magnetocaloric material’s temperature or entropy change can be harvested cyclically to pump heat from one reservoir (the cold sink) to another (the heat sink). The chief advantages of magnetic refrigeration versus current gas compression technologies are (i) the absence of gases with high global warming potential, (ii) the potential to reach higher Carnot efficiency, and (iii) the reduction of noise and maintenance.^1,2^ As a result, this technological potential has attracted numerous key industrial players.^3,4^

Over the past few decades, researchers discovered several magnetocaloric compounds. In particular, those that exhibit the so-called giant magnetocaloric effect, such as Gd_5_Si_2_Ge_2_,^5−7^ La(Fe,Si)_13_-based alloys,^8,9^ Heusler alloys,^10−13^ Fe–Rh,^14^ Ni–Mn–Ga,^15^ and Mn–As,^16^ have attracted much attention. From this list, the performance and the less-critical (more abundant) elements that make up La-based alloys, in particular when compared to Gd-based and other critical raw-element-based compounds, has singled out La(Fe,Si)_13_ as one of the most promising families of magnetocaloric materials. Various researches have been reported on the engineering, control, and optimization of the magnetocaloric properties of these substances,^17^ including the partial substitution of Fe by other elements, such as Co^18,19^ and Mn,^8^ as well as introducing of interstitial H and C atoms.^20^ Importantly, interstitial hydrogenation can lead to a shift in the Curie temperature of such materials toward room temperature, while retaining their large magnetocaloric effect.^8^

A review of the anticipated applications for magnetocaloric systems suggests that lightness (or portability), mechanical and chemical stability, flexibility, ease of processing, and tunable geometries are key properties that will facilitate implementing these materials in real life refrigerators and waste heat energy harvester devices.^21−26^

Various methods have been proposed to control the properties of the abovementioned materials by mixing them in different composites within the polymer or metal matrices. Metal-bonded magnetocaloric composites have been methodically investigated in the literature.^27−29^ The use of ductile metals with low melting point and good thermal conductivity as binders, such as Cu, Ti, and In, is considered a promising approach that can improve the composite thermal conductivity.^30−32^ However, these metal-based compounds are hard to shape, typically require high temperature and costly processing procedures, and employ a large mass of inactive material (the metal matrix). Alternatively, polymer-bonded magnetocaloric composites enable composite shaping in a wide variety of forms and typically permit low-cost processing and a much lower mass of inactive material (the polymer matrix), despite their typically low thermal conductivities. In particular, polymer-based magnetocaloric composites produced by adhesive bonding can improve corrosion protection and mechanical stability and produce net-shaped modules in a single step. Radulov et al. performed a thorough study on the magnetocaloric properties of adhesive-type polymer-bonded La(Fe,Mn,Si)_13_H_*x*_, in which samples made by normal epoxy not only promote higher Δ*S*_m_ and Δ*T*_ad_ but also lead to improved mechanical properties.^11^

Furthermore, the smart design of magnetocaloric materials with functional polymers enables the development of multifunctional composites exhibiting large responses to different external stimuli (including magnetic, electric, and pressure fields) and cross-coupling effects, such as magnetoelectric coupling in multiple systems.^33−35^ Within the context of caloric applications, these systems are known as multicaloric composites.^34,36−38^ The application of multicaloric materials in prototype devices has already enabled reaching higher cooling power^39^ and efficiency.^40^ A prime example of the latter is the multicaloric heat pump design proposed by Gottschall et al. that makes use of the barocaloric and magnetocaloric effects by applying/removing uniaxial stress and magnetic fields, respectively, in such a way that hysteresis is further exploited.^41^

To develop multicaloric/multifunctional composites, micro- or nanostructuring of the magnetocaloric materials is vital. Typically, researchers employ either a top-down or bottom-up approach. The design of polymeric composite films with magnetocaloric inclusions using solvent-casting techniques has led to promising results.^21,42^ In particular, an interesting report on magnetocaloric microparticles embedded in the poly(methyl methacrylate) matrix showed that the thermoplastic polymer could affect the particles, acting as a pressure cell and leading to a reduction of the magnetocaloric effect of the system.^21^ Concerning polymers, poly(vinylidene fluoride) (PVDF) is a well-known EA polymer that has different crystallographic phases (α, β, γ, δ, and ε) influenced by C–H–F formation.^42^ EA polymers respond to changes in the electrical input. The β and γ phases are more desirable considering their EA and piezoelectric nature, while the α and ε phases are nonpolar. These properties lead to additional applications for PVDF-based composites including actuators, sensors, and batteries. Amirov et al. reported a multiferroic composite based on magnetic Heusler-type microwires embedded in a PVDF matrix using a modified solvent-casting technique. They claimed the existence of magnetocaloric and magnetoelectric effects peaking around the phase transition temperature, suggesting the potential development of new smart materials.^43^

Sasmal et al.^49^ employed the solvent-casting technique to fabricate composite films based on PVDF with varying concentrations of YFeO_3_ powder. The PVDF film loaded with 5 wt % YFO displayed a substantial polar phase percentage of approximately 85% allowing mechanical energy harvesting. In addition, the application of an external magnetic field modulated the mechanical energy harvesting capabilities, suggesting potential applications in self-powered magnetic field sensing.^44^

PDVF-based composites with Gd_5_(Si,Ge)_4_ magnetocaloric microparticle inclusions, fabricated by the casting method, were recently studied by Andrade et al. with the objective of unlocking the multicaloric potential of such composites.^42^ However, uniform dispersion of inclusion particles in the polymer solution has always been a major challenge in the casting method.^45,46^

In this work, we take an alternative approach, using the electrospinning technique to make flexible polymer composite mats using PVDF nano/microfibers and La(Fe,Si)_13_-based microparticles. Electrospinning is a low-cost and relatively simple technique to produce nonwoven mats with fiber diameters ranging from a few nanometers up to several micrometers.^47,48^ With electrospinning, nanofiber production can be easily scalable to large volumes and sizes for a variety of applications.

The present research focuses on fabricating and characterizing magnetocaloric fiber mats, aiming to encapsulate LaFe_11.83_Mn_0.32_Si_1.28_H_*x*_ particles inside PVDF polymer fibers to form a flexible, easily shaped, mechanically resistant to bending fatigue, and freestanding mat, while retaining the LaFe_11.83_Mn_0.32_Si_1.28_H_*x*_ magnetocaloric properties and preventing their corrosion. By taking advantage of the unique magnetic properties of La(Fe,Si)_13_ when embedded inside the electroactive/piezoelectric polymer fiber mats, we have developed flexible and multifunctional nano/microfibers with potential applications in multicaloric heat-pumping devices and other sensing and actuating devices.

## 2 Experimental Section

### 2.1 Sample Preparation

The LaFe_11.83_Mn_0.32_Si_1.28_H_*x*_ powder, which will be referred to as LaFeSi for simplicity, was prepared by arc-melting, followed by hydrogenation.^11^ After melting, to ensure homogeneity of the as-cast alloy, we encapsulated the ingot in quartz tubes under an Ar atmosphere and then annealed it at 1050 °C for 7 days, with a subsequent quench in water. 1–2 mm fragments of the parent bulk sample were hydrogenated in a furnace filled with a 0.9 bar H_2_ atmosphere, at 450 °C for 1 h, to saturate the H concentration. The as-prepared hydrogenated pieces of LaFeMnSiH were hand-milled for a long period and then sorted with a 10 < *d* < 50 μm sieve. This step led to the collection of powder with a maximum particle size of 50 μm.

Flexible fiber mats based on PVDF with and without the LaFeSi filler, 0.05 g ∼ 10 wt % of polymer mass, were prepared by an electrospinning technique. The PVDF mat with LaFeSi particles is hereafter referred to as PVDF-LaFeSi. The PVDF precursor solution results from a combination of PVDF-5130 (*M* > 1,000,000 g/mol, acquired from Solef) in dimethylformamide (DMF) solvent (Sigma), to achieve a solution with a concentration of 0.105 g/L. After several preliminary experiments, we found this concentration to have an optimal specific viscosity leading to the formation of bead-free and aligned fibers by a stable electrospinning process, while preventing the spray effect.

In order to achieve a uniform mixing, we stirred the initial PVDF solution overnight with a magnetic stirrer at room temperature. Then, considering the ferromagnetic nature of the LaFeSi powder at room temperature, we raised the solution temperature up to ∼30 °C (above LaFeSi Curie temperature (*T*_c_) ∼ 22.8 °C), in order to promote a random particle dispersion. Mechanical stirring was the last treatment, which helped to achieve a homogeneous suspension of LaFeSi nanoparticles in the initial PVDF/DMF solution before electrospinning. Intense mechanical stirring followed by a prolonged sedimentation time resulted in a naturally occurring selection of the smaller particles present in the solution.

**Figure 1:**
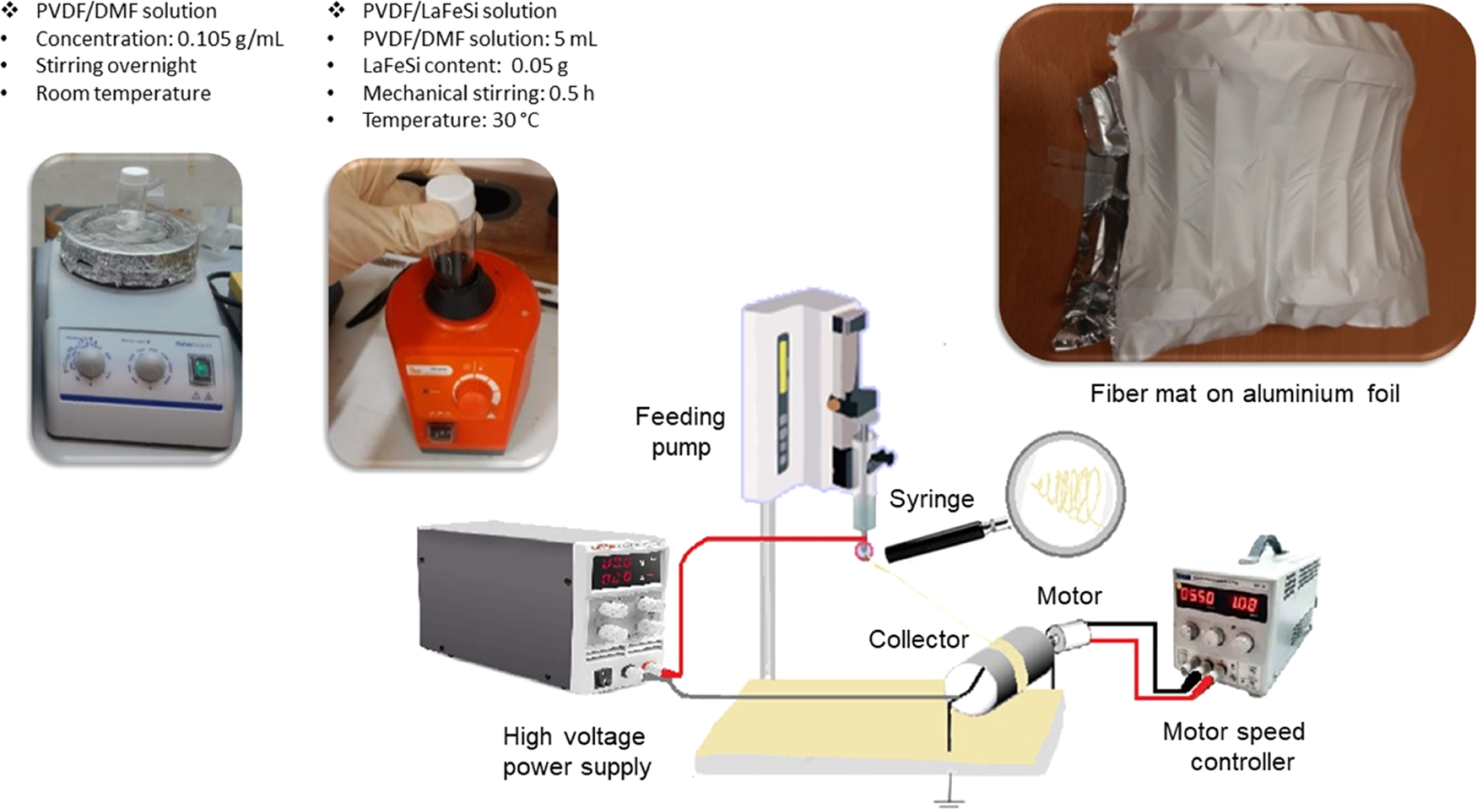
Schematic illustration of fiber mat preparation along with the photograph of the as-obtained mat over aluminum foil.^47^

We prepared two different nano/microfiber mats by electrospinning: PVDF mat and PVDF-LaFeSi mat. We used the following parameters adjusted to optimize the electrospinning process. A 5 mL syringe containing the as-prepared solution was attached to a 0.6 mm diameter needle, and a DC voltage of 18 kV was applied between the needle and a grounded rotatory collector. We set the distance between aluminum foil, used as the grounded collector of the fibers, and the needle tip at 11 cm and a flow rate at 0.20 mL/h using a syringe pump. The electrospinning process was carried out under ambient conditions. Figure 1 summarizes the synthesis procedure for the electrospun fiber mats.

### 2.2 Characterization Techniques

Micrographs of the electrospun fiber mats were obtained using an ultrahigh resolution field emission gun scanning electron microscope (FEG-SEM), NOVA Nano SEM 200, FEI Company. This was done under different magnifications (5000 and 50,000×) with an energy of 15 kV and in different modes, namely, secondary electron (SE) and backscattered electron contrast. In order to enhance the sample electrical conductivity, 15 nm thick Au–Pd (80–20 wt %) films were deposited on the samples surface using a high-resolution sputter coater, model 208HR, from Cressington, coupled to a MTM-20 Cressington high-resolution thickness controller. The histograms of the fiber diameters were obtained using ImageJ 1.46r image analysis software, applied to extended sample areas of the FEG-SEM images. As a complementary technique, the energy-dispersive X-ray spectroscopy (EDS) analysis evaluated the chemical composition of the mats, using an EDAX Si (Li) detector at an acceleration voltage of 15 kV.

X-ray diffraction (XRD) was used in order to characterize the structure of the fiber mats and that of the initial powders, for 2θ angles between 20 and 70° and at room temperature. A Rigaku Smart Lab diffractometer, with Bragg–Brentano geometry and a Cu-k_α_ radiation source, with a wavelength of λ = 1.5406 Å, was used for this analysis. When possible, the patterns were analyzed using FullProf Suite Software.^49,50^

Attenuated total reflection Fourier transform infrared (ATR-FTIR) spectroscopy was carried out at room temperature in the wavenumber range of 1450–450 cm^–1^ on a PerkinElmer Spectrum BX spectrophotometer equipped with an ATR accessory, with 2 cm^–1^ resolution.

Atomic force microscopy (AFM) measurements, local piezoresponse, and local polarization switching spectroscopy of the fibers made by employing the piezoresponse force microscopy (PFM) method were performed on a Veeco Multimode Nanoscope IV microscope (MMAFM- 2, Michigan Tech, Houghton, MI) using the conductive probe (NSG10/Pt, NT-MDT). A spring constant of 14 N/m was used, and the PFM out-of-plane images were scanned in the single frequency PFM mode at 35 kHz and 7.5 V. The magnetic force microscopy (MFM) scans were obtained using the second-pass technique at a lift height of 50 nm. Commercial cantilevers coated with a magnetic layer of CoCr (MFM01, TipsNano, tip apex radius 45 nm; tip coercive field (*H*_C_) > 5 kOe) were utilized for the measurements.

A Quantum Design MPMS 3 superconducting quantum interference device (SQUID)-based magnetometer was used to study the sample’s magnetic properties under applied magnetic fields up to ∼1.9 T and within the 270–330 K temperature range. In particular, the isothermal entropy changes of the composite fiber mat and the individual magnetocaloric powder were estimated from isothermal magnetization versus applied magnetic field curves. Additionally, direct measurements of the adiabatic temperature change were carried out in an experimental setup based on two nested Halbach cylinders with a uniform magnetic field perpendicular to the cylinder axes. Mutual rotation of the cylinders resulted in a harmonically varying magnetic field of ±1.93 T. The frequency used in the experiment was 0.05 Hz, corresponding to a maximum field sweep rate of 0.5 T/s. The setup is described in detail elsewhere.^51,52^

## 3 Results and Discussion

### 3.1 Morphological and Elemental Characterization

**Figure 2:**
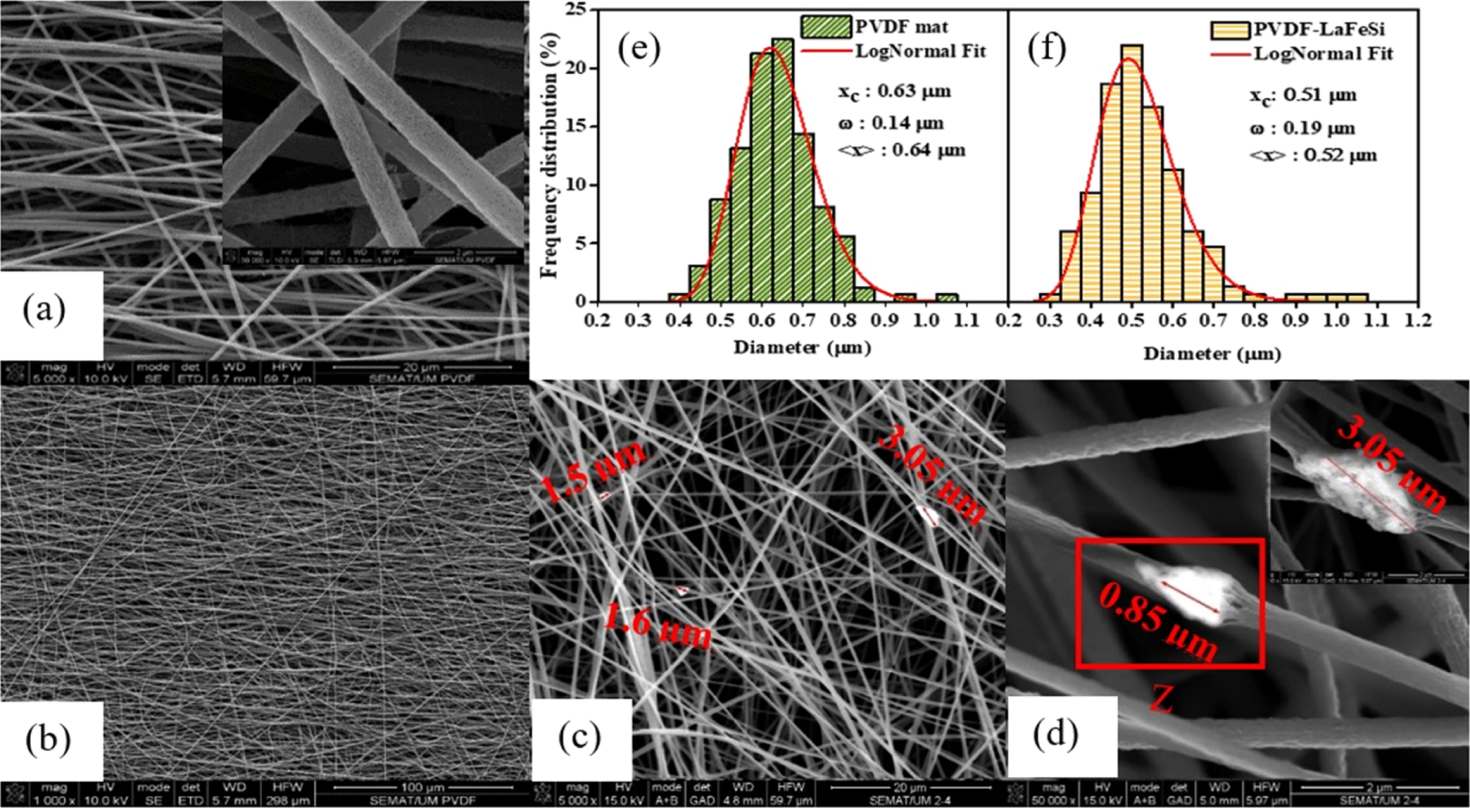
FEG-SEM images of (a, b) pure PVDF (topographic mode with SE detector) and (c, d) PVDF-LaFeSi fiber mats (contrast mode with GAD detector), with different magnifications. (e, f) Histogram of the diameter distribution of pure PVDF and PVDF-LaFeSi fibers.

The morphology of the fiber mats was investigated by using FEG-SEM analysis. Figure 2a–d depicts images of the PVDF mat as well as of the PVDF-LaFeSi composite fibers, at different magnifications. As can be seen, the fibers are relatively uniform and straight over extensive lengths (up to hundreds of micrometers). The LaFeSi magnetic microparticles are well dispersed and well incorporated inside the PVDF fibers.

The distribution of the diameters of PVDF and PVDF-LaFeSi fibers was evaluated using the ImageJ 1.46r image analysis software over a 300 μm^2^ of the fiber mat’s area. A fit with the log-normal function , where *x*_c_ and ω are fitting parameters) allows us to estimate the average diameter, ⟨*x*⟩, and the distribution width, ω, of the fibers. For this distribution, eq 1 gives the average diameter.
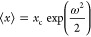
1

Figure 2e–f presents the histograms of the diameter distribution for the fiber mats. Based on this study, 0.64 and 0.14 μm were the average diameter and distribution width, respectively, for the pure PVDF fibers, and 0.52 and 0.19 μm for the PVDF-LaFeSi mat.

The EDS spectrum of the magnetic particles within the polymeric fiber matrix highlighted as Z in Figure 2d is presented in the Supporting Information, Figure S1. As anticipated, the EDS spectrum shows C and F arising from PVDF and La, Fe, Mn, and Si from the inclusion of LaFe_11.83_Mn_0.32_Si_1.28_H_*x*_. There are also some small peaks from Pd and Au due to the sputtered conductive thin film of Au–Pd on top of PVDF and PVDF-LaFeSi fiber mats, required for FEG-SEM imaging. The corresponding atomic percentages (atom %) of the incorporated particle elements are shown in the inset of Figure S1. Ratios of Si/La, Mn/La and Fe/La indicate that the experimental results are in reasonable agreement with the nominal values, pointing to a preservation of the chemical composition of the magnetic microparticles located inside the polymer fiber mat.

### 3.2 Structural Characterization

**Figure 3:**
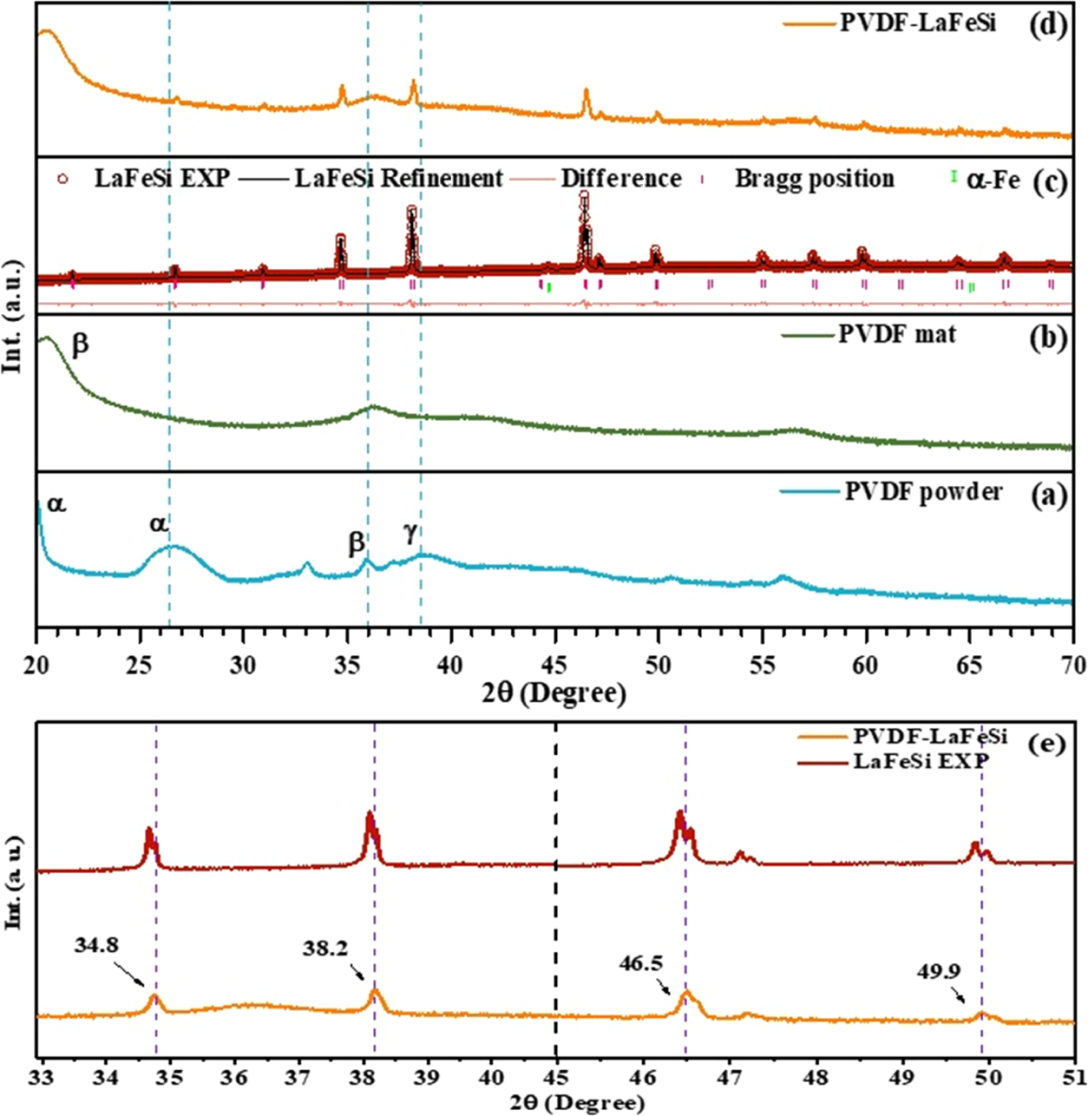
XRD patterns of (a) PVDF powder, (b) PVDF mat, and (c) LaFeSi powder: experimental data (red open circle), Rietveld refinement curve (black line), difference pattern (pink line), Bragg positions of LaFeSi powder (pink ticks) along with α-Fe phase (green ticks), and (d) PVDF-LaFeSi mat. (e) Zoom-in on the 2θ range of ∼33 to 51° of the X-ray diffraction results.

X-ray diffraction patterns were obtained for the PVDF mat and for the PVDF-LaFeSi fiber mats as well as for pure PVDF powders and LaFeSi (before and after dispersion in DMF) precursors. The diffractograms are presented in Figures 3a–d and S2. With the PVDF powder, the most intense peak observed for 2θ < 20° and the broader one at 2θ ∼ 26° correspond to the α phase and refer to the corresponding (110) and (021) atomic planes, respectively.^53^ The broad peaks at 2θ ∼36 and ∼38.5° are associated with the (201) and (211) atomic planes of the β and γ crystalline phases of PVDF, respectively.^53−56^ All PDVF powder diffraction peaks present a broad and bumpy form, revealing their nanocrystalline structure with nanometer scale crystals. For the PVDF mat (Figure 3b) the peak at 2θ > 20° refers to the superposition of the peaks associated with the β (110) and β (200) planes^54,57,58^ indicating that the stretching of the polymer jet during electrospinning favors the formation of polar phases of PVDF.^54^ The peak at 2θ ∼ 36° also appeared as a bump in the XRD pattern of the PVDF fiber mats. Rietveld refinement was performed on the diffractogram of the LaFe_11.83_Mn_0.32_Si_1.28_H powder, and the result is presented in Figure 3c (Rietveld refinement parameters: *R*_p_: 1.51, *R*_ωp_: 2.34, and *R*_exp_: 0.02). The Rietveld refinement shows that LaFeSi grains crystallized in a cubic NaZn_13_-type structure with a minor amount of α-Fe phase (∼3%) as typically occurs in these materials.^20,59,60^ The estimated volume and lattice parameters are 1546.115 (±0.006) Å^3^ and 11.56327 (±0.00003) Å, respectively, and they are in agreement with the literature.^61^ It is important to remark that XRD was performed at room temperature (∼297 K), that is, at a temperature above LaFeSi’s *T*_c_. Therefore, the compound was in the paramagnetic state and its unit cell was in the low-volume state.

Comparing the XRD pattern of the PVDF-LaFeSi fiber mat with that of the pure LaFeSi powder, in the 2θ range of ∼33 to 51° (Figure 2e) confirms the presence of the peaks corresponding to the LaFeSi inclusions inside the PVDF-LaFeSi mat. The absence of additional peaks represents an important check for the absence of any additional secondary phases that could have formed during the composite processing.

### 3.3 ATR-FTIR Analyses

**Figure 4:**
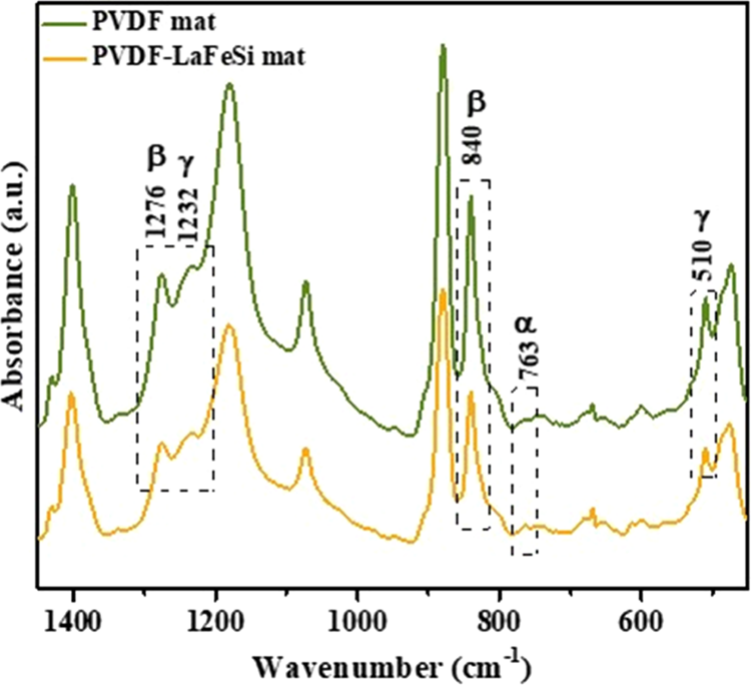
FTIR-ATR spectra of PVDF and PVDF-LaFeSi mats.

When there is more than one electroactive phase in the material, as is often the case with PVDF, ATR-FTIR is a useful technique to evaluate the contributions of the different phases. Several studies have been done in which the infrared peaks of different PVDF phases are reported.^62,63^Figure 4 presents the ATR-FTIR spectra that were collected for the PVDF mat and PVDF-LaFeSi composite fiber mat in the 1450–450 cm^–1^ wavenumber range at room temperature. According to the extended FTIR research by Cai et al.,^62^ both the 1276 and 1232 cm^–1^ bands appearing together in the ATR-FTIR spectrum and the bands at 840 and 510 cm^–1^ are characteristic of a combination of the β and γ electroactive phases of PVDF. The results then show that both samples are mainly composed of a mixture of β and γ phases, attested by the sharp individual peaks belonging to each of those phases. Additionally, the spectrum of the fiber mats shows a low-intensity peak at 763 cm^–1^. This peak is typically attributed to the presence of a residual α phase, the nonelectroactive phase of PVDF, as often mentioned in the literature.^63−65^

### 3.4 Local-Scale PFM and MFM Characterization

**Figure 5:**
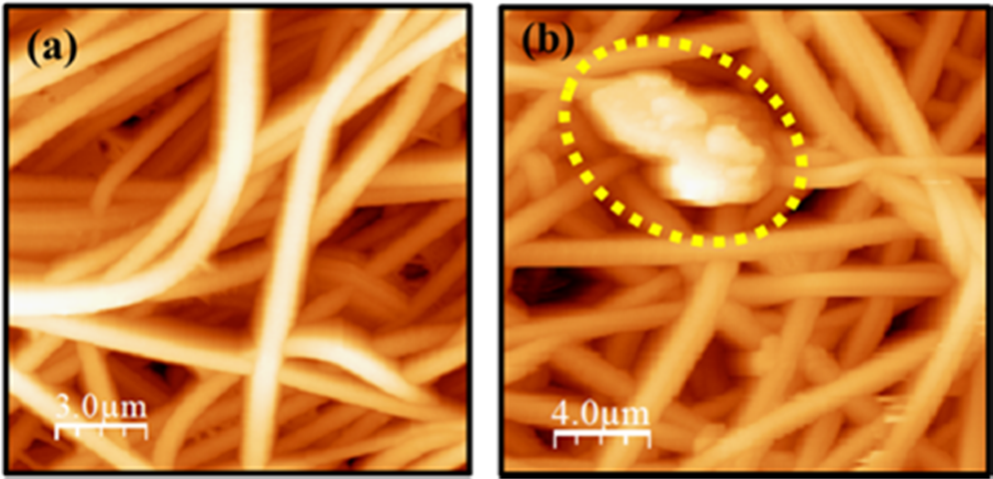
AFM topography image of (a) PVDF and (b) PVDF-LaFeSi mats.

The local-scale piezoelectric and magnetic responses of the electrospun fiber mats were investigated by PFM and MFM techniques, respectively. Figure 5a,b displays the AFM topography images of the pure PVDF and PVDF-LaFeSi fiber mats. The presence of LaFeSi particles is shown in Figure 5b. The fiber morphology observed in the AFM images is similar to that identified by FEG-SEM. Analysis of height maps reveals average diameters of ∼0.79 and ∼1.09 μm for the pure PVDF and PVDF-LaFeSi fibers, respectively. The slight discrepancy with SEM values results from the different areas probed by the two techniques. The average roughness (RMS roughness) of the fiber surface was found to increase from 0.65 μm in pure PVDF to 0.76 μm in PVDF with LiFeSi filler. This increase in roughness could be a result of irregular stresses arising during the crystallization of the fibers owing to the presence of distributed LiFeSi particles having different average sizes.

**Figure 6:**
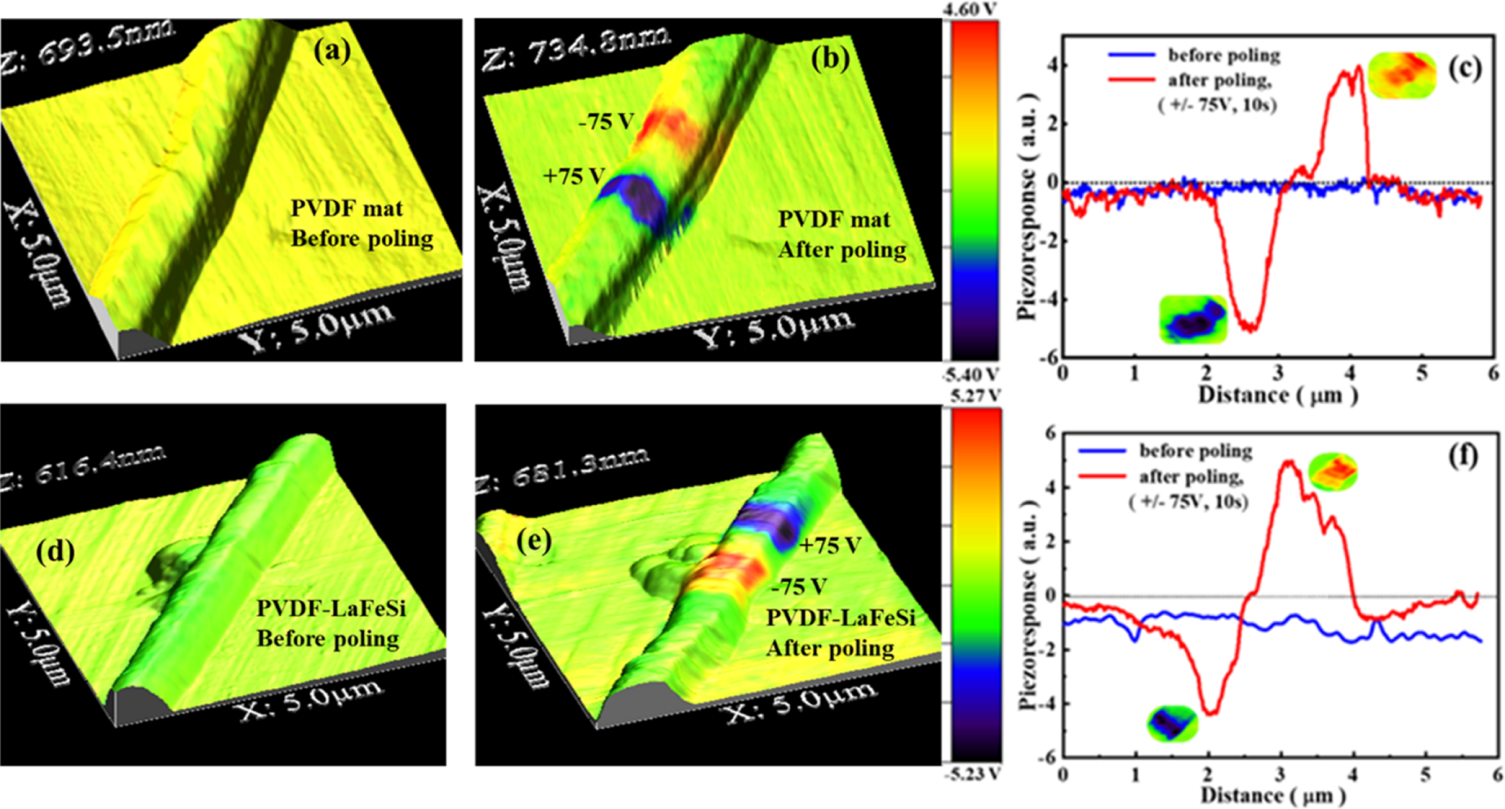
PFM scans (superimposed out-of-plane PFM and topography) of individual fibers under (a, d) unpoled states; (b, e) artificially written domain (poled with ±75 V); and (c, f) profiles of domains. Top and bottom panels present pure PVDF and PVDF-LaFeSi, respectively.

The piezoelectric properties of the fiber mats were determined from the local piezoresponse and hysteresis loops derived from the PFM studies. Figure 6 shows the PFM micrographs obtained on a single fiber in each mat. The respective fibers did not show any ferroelectric domains in the virgin state (Figure 6a,d). However, the PFM scans after the application of a *dc* bias voltage of ±75 V for 10 s revealed the tip-induced polarization regions or the artificially written domains within the fiber, as shown in Figure 6b,e. By comparing the unpoled and poled PFM micrographs, it is clear that there is an absence of direct correlation between the piezoelectric contrast and topographic features, thereby indicating that the observed effect originates from the intrinsic piezoelectric property of the fibers and not from electrostatic effects and/or cross-talk with topography.^66^ To understand the effect of the induced polarization, the piezoresponse signals obtained before and after poling across the PVDF and PVDF-LaFeSi fibers were plotted, as shown in Figure 6c,f, respectively. With the PVDF-LaFeSi composite fibers (Figure 6f), the negative voltage polarity leads to an enhanced piezoelectric response. Also, the PVDF-LaFeSi fiber exhibits a higher negative piezoresponse in the unpoled condition, which is indicative of a self-polarized state. It is noteworthy that we used aluminum foil as the substrate, which has a more negative potential than the cantilever, so a negative-shift effect can be expected. In both samples, we can see a negative shift. However, it is higher for the PVDF-LaFeSi sample. This indicates that, due to the inclusion of LaFeSi in the PVDF, either a local alignment of domains or excess negative charge resulted in a local self-polarization before poling.

We estimated the size of the domains for the respective voltage polarity as follows: 0.67 μm (positive) and 0.59 μm (negative) with PVDF, while for PVDF-LaFeSi, it is 0.87 μm (positive) and 1.56 μm (negative). The domains in PVDF-LaFeSi are bigger than that in the PVDF mat, which is also apparent in the wider peak for PVDF-LaFeSi (Figure 6f). It is the presence of conductive filler particles that created favorable conditions for the propagation of the electric field, resulting in a larger domain size in the PVDF-LaFeSi composite fibers.

**Figure 7:**
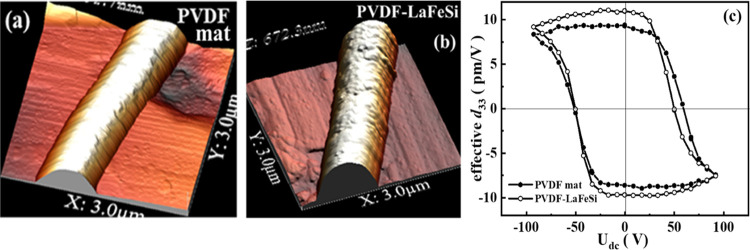
3D topography image of the single fibers of (a) pure PVDF fibers and (b) PVDF-LaFeSi fibers and (c) comparison of loops between PVDF and PVDF-LaFeSi fibers.

To study the piezoelectric domain switching behavior, we acquired the hysteresis loops on a single fiber that were measured in the pulse mode by sweeping the *dc* bias voltage (*U*_dc_) between ±90 V, as well as ±75 V. The hysteresis loop for voltage ±90 V is shown in Figure 7c, while the loop obtained at ±75 V is available as Figure S3. The obtained PFM loops indicate the existence of ferroelectricity (local switching process with domain nucleation and switching under the tip) in the fibers.

The electrospun fibers show an effective longitudinal piezoelectric coefficient *d*_33_ = −11.01 pm/V in PVDF-LaFeSi, which is higher than that of the pure PVDF fibers (−9.36 pm/V). The effective *d*_33_ value is higher and/or comparable to those reported in the literature for micrometer size PVDF fibers.^66−70^ A possible reason for the higher effective *d*_33_ in PVDF composite fibers (PVDF-LaFeSi) is the static electric interactions between the CH_2_ groups of PVDF and the LaFeSi magnetic particles that can effectively induce nucleation of the polymeric electroactive phase of PVDF.^71^ Considering the average thickness of the fibers, the estimated coercive field (*E*_c_) is ∼59.85 MV/m for pure PVDF and ∼50.61 MV/m for PVDF-LaFeSi, which are well within the range of reported values for PVDF.^72,73^

**Figure 8:**
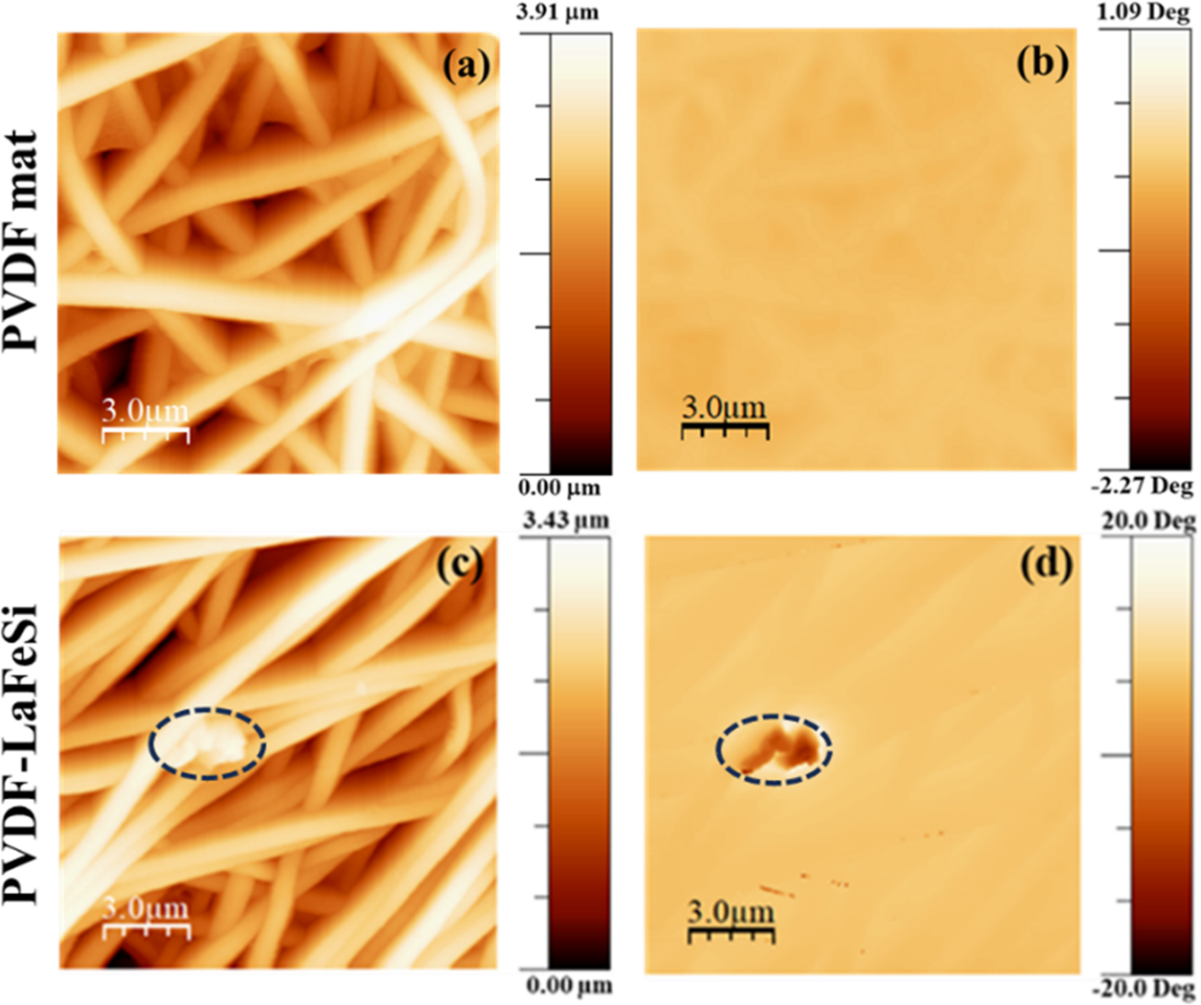
(a, c) Topography image and (b, d) MFM image of the pure PVDF and PVDF-LaFeSi mats.

To elucidate the magnetic behavior of the LaFeSi filler at the nanoscale, MFM was carried out. Figure 8 displays the MFM scans (15 × 15 μm^2^) of the fiber mats under a zero external magnetic field. As seen, the pure PVDF polymer matrix shows no magnetic signal, while a magnetically active region of ∼2.9 μm is observed in the composite sample, confirming the presence of the magnetic LaFeSi particles in the polymer matrix.

### 3.5 Magnetic Characterization

**Figure 9:**
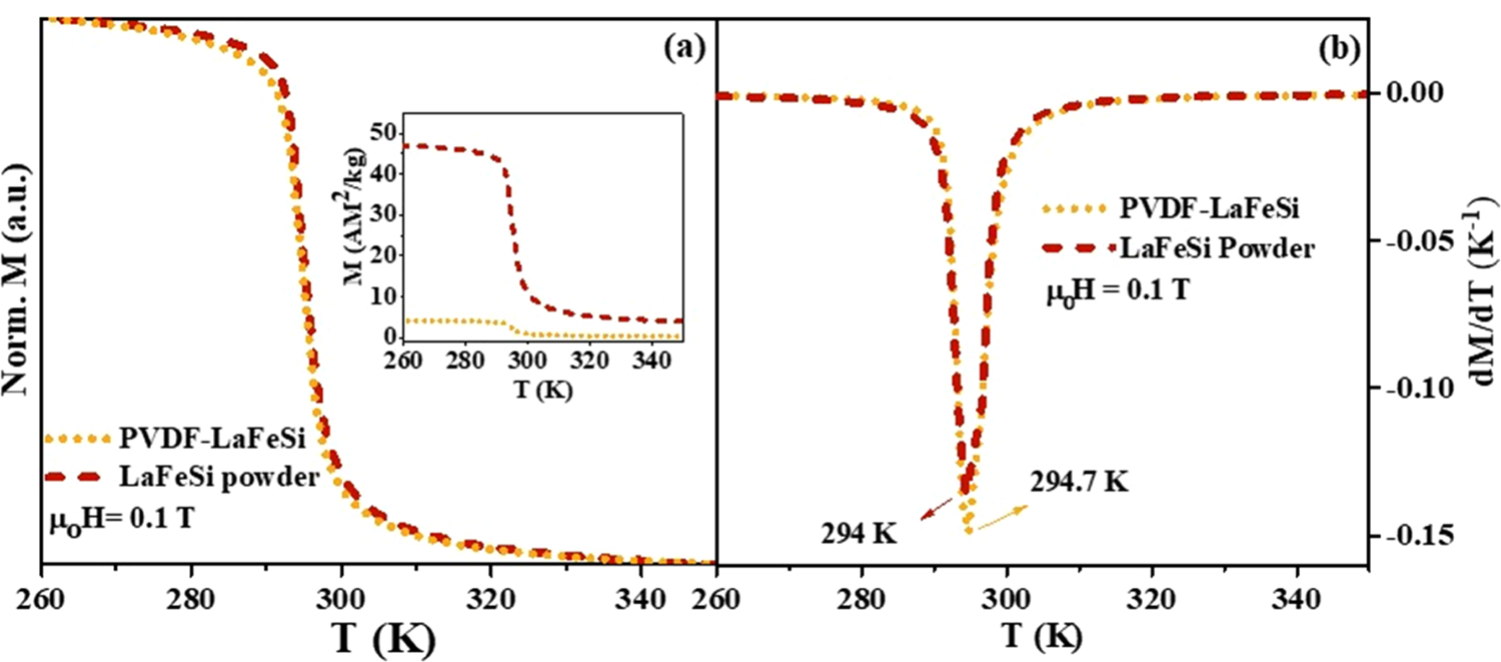
(a) Temperature dependence and (b) temperature derivative curves of normalized magnetization regarding pure LaFeSi powder and PVDF-LaFeSi composite fiber mats under the magnetic field of 0.1 T. The inset of panel (a) shows magnetization versus temperature, without normalization.

The temperature dependence of the PVDF-LaFeSi composite and LaFeSi powder magnetizations were studied under a 0.1 T applied external magnetic field, in the temperature range between 260 and 350 K. As shown in Figure 9a, the normalized magnetization versus temperature curves (*M*(*T*)/*M*_*T*=260K_) of the PVDF-LaFeSi composite and LaFeSi powder samples are quite similar. A drastic change in magnetization is seen near 300 K. This sharp change, which is more obvious from curves of the temperature derivative of magnetization, shown in Figure 9b, is due to the para to ferromagnetic (PM-FM) phase transition of LaFeSi, under cooling, which occurs at *T* = *T*_c_ = 294 K and has a slight shift to 294.7 K for the composite sample. This *T*_c_ value is in agreement with that reported for bulk La(Fe,Mn,Si)_13_H_*x*_.^11^

### 3.6 Magnetocaloric Analysis

**Figure 10:**
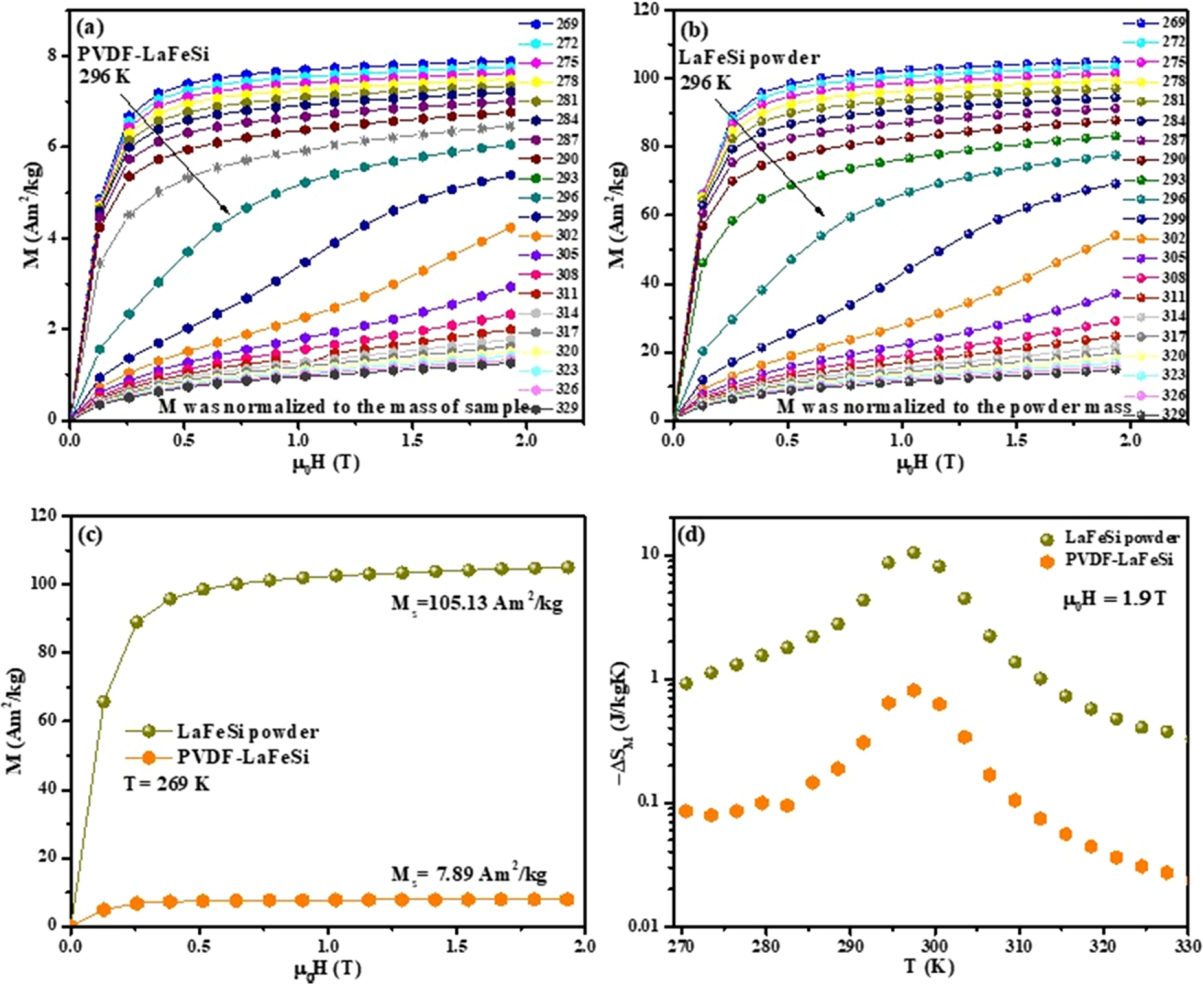
(a, b) Isothermal field dependence of magnetization, *M*(*H*)*_T_*, loops, (c) comparison between the *M*(*H*) loops at 269 K, and (d) isothermal magnetic entropy change under an applied field change of 1.9 T, Δ*S*_M_, recorded for PVDF-LaFeSi composite fibers (orange) and LaFeSi powder (green). 3 K was chosen as the temperature interval between the *M* (*H*)*_T_* sequences of measurements.

The magnetization isotherm curves, *M*(*H*)*_T_*, for the LaFeSi powder and the PVDF-LaFeSi composite mat were recorded from 269 to 329 K, with a 3 K step. These magnetization isotherms are presented in Figure 10a,b. It should be noted that for the PVDF-LaFeSi sample, the total measured magnetic moment was normalized to the total mass of the sample. The saturation magnetization values of the PVDF-LaFeSi composite fiber mat and LaFeSi powder, at 269 K, are 7.89 and 105.13 Am^2^/kg, respectively, according to Figure 10c. Here, the dilution of the LaFeSi powder in the nonmagnetic PVDF polymer matrix leads to a reduction in the saturation magnetization. However, comparing the PVDF-LaFeSi experimental saturation magnetization with the one expected for the composite based on the LaFeSi nominal concentration, the amount of LaFeSi powder involved in the electrospinning process is lower than the nominal concentration of ∼10 wt %, as already noted in Section 2. Based on the results of Figure 10a,b, only about 7.5 wt % (*M*_s_fibers_/*M*_s_powder_ = 7.89/105.13) of magnetocaloric powder was incorporated in the fibers during the electrospinning process. This is consistent with our observations during the fiber mat preparation since a significant portion of the powder remained inside the syringe, mainly composed of the larger LaFeSi particles.

The similarity between the magnetization curves under increasing and decreasing field modes in the M(H) loops demonstrates the effective magnetic reversibility and consequent absence of significant hysteretic losses. Moreover, the sharp change in the magnetization, which is more obvious above 296 K, is a signature of a first-order magnetic phase transition.^74^ This transition is particularly clear beyond a critical field. The critical field shifts toward higher values while increasing the temperature, which is also a signature of the typical first-order itinerant electron metamagnetic (IEM) transition. This behavior is known for La(Fe,Si)_13_-based compounds with the cubic NaZn_13_-type (1:13) structure.^75^ In fact, the field-induced metamagnetic transition from the PM to FM state at temperatures near but above *T*_c_ has been observed for LaFe_13–*x*_Si_*x*_ compounds with *x* < 1.6.^75^ In order to quantify the magnetocaloric effect (MCE) of the samples, the isothermal magnetic entropy change, Δ*S_M_*, and the adiabatic temperature change, Δ*T*_ad_, were measured as a function of the magnetic field and temperature.

Figure 10d shows the isothermal magnetic entropy change, Δ*S_M_*, for PVDF-LaFeSi fibers and LaFeSi powder. Δ*S_M_* was calculated from the *M*(*H*)*_T_* dependence using the Maxwell relation, presented in eq 2. Δ*S*_*M*_^up^ was evaluated under an increasing externally applied magnetic field, in the range of 0 to ∼1.9 T.
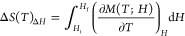
2The Δ*S_M_*(*T*,Δ*H*) curve profiles reach their peak values, Δ*S*_*M*_^max^, near *T*_c_, and the peak is asymmetric. Figure 10d shows values of 15.7 and 1.2 J/kgK for Δ*S*_*M*_^max^ of the LaFeSi powder and of the PVDF-LaFeSi composites, respectively. Consistent with the saturation magnetization analysis, the ratio between the Δ*S*_*M*_^max^ values of the PVDF-LaFeSi composite and LaFeSi powder is equal to 7.8%.

In addition, considering 3 K as the temperature interval between consecutive measurements, there is no significant shift in transition temperature for the PVDF-LaFeSi composite, compared to the LaFeSi powder, revealing no major influence from the ferroelectric polymer on the filler intrinsic magnetic properties, which is also in agreement with Figure 9a,b.

The broad form of the Δ*S* versus temperature curve enhances the temperature range of the heat transfer between the cold and hot sides and, as a result, improves the cooling efficiency as a practical parameter for evaluating the potential of cooling systems and device design. This broad temperature behavior gives a wide working temperature interval of ∼30 K around room temperature, which is important for applications in magnetic refrigeration or waste heat energy harvesting near ambient temperature.

**Figure 11:**
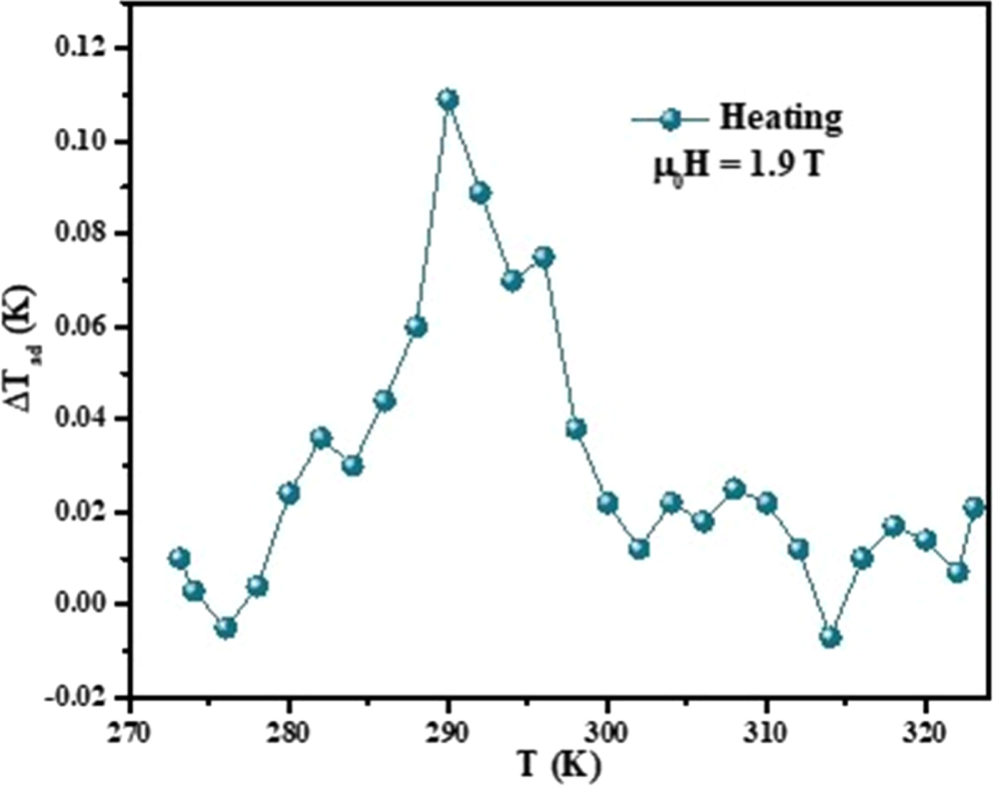
Adiabatic temperature change Δ*T*_ad_ as a function of temperature.

As a complementary study, Δ*T*_ad_ direct measurements were also performed with magnetic field changes of up to 1.9 T. The measurements were performed in the 273–323 K temperature interval. Figure 11 shows the temperature dependence of the adiabatic temperature change, Δ*T*_ad_, for the PVDF-LaFeSi mat during heating. The magnetic field change was Δμ_0_*H* = 1.9 *T*. The figure shows that Δ*T*_ad_(*T*) reaches its maximal value of 0.11 at 290 K, with a broad width of the peak near room temperature. By increasing the amount of the LaFeSi phase inside the composite fibers, further increases in Δ*T*_ad,_ can be expected.

## 4 Conclusions

The variety of applications and requirements of cooling systems has caused many researchers to turn to this topic, in particular, to the study of magnetocaloric materials for refrigeration. In the present research, LaFe_11.83_Mn_0.32_Si_1.28_H_*x*_ powder (LaFeSi) was prepared by arc-melting, followed by a hydrogenation procedure and then incorporated into a polymeric fiber matrix (PVDF-LaFeSi). The objective was to obtain a flexible magnetocaloric material with enhanced performance at around room temperature. X-ray diffraction, electron microscopy, and infrared spectroscopy confirmed the production of composite fibers with well-formed LaFeSi particle inclusions inside the PVDF polymeric matrix. The PFM and MFM results showed the presence of a ferroelectric phase in PVDF along with the ferromagnetic phase of the LaFeSi inclusions.

On the other hand, including these powders inside a PVDF polymeric fiber mat, using electrospinning, demonstrated to be a simple and cost-effective technique to induce significant MCE, Δ*S*_M_^max^ values (1.2 J/kgK for the PVDF-LaFeSi composite) using very low content of LaFeSi. The observed extended working temperature interval of ∼30 K around room temperature for the PVDF-LaFeSi composite nano/microfiber makes this material a potential candidate for magnetic refrigeration/waste heat energy harvesting near ambient temperature.

On the other hand, the effective longitudinal piezoelectric coefficients (*d*_33_) for the PVDF-LaFeSi and PVDF fiber matrix were −11.01 and −9.36 pm/V, respectively. Combining the magnetic properties of the LaFeSi grains and the polar properties of the PVDF matrix helps to promote a magnetoelectric coupling. As a result, these flexible polymer mats using polar PVDF nano/microfibers and La(Fe,Si)_13_-based magnetic particles as fillers have the potential to be used as environmentally friendly and flexible materials for light and miniaturized magnetocaloric refrigeration and magnetoelectric devices.

## Supporting Information Available

EDS spectrum of a LaFeSi particle inclusion, the XRD spectra of the LaFeSi powder before and after dissolution in DMF, and PFM hysteresis loops of PVDF and PVDF-LaFeSi fibers (PDF)
