# Supplementary material for: Flexible Magnetocaloric Fiber Mats for Room-Temperature Energy Applications
Source: ACS Appl Mater Interfaces. 2024 Feb 1;16(7):8655–67. doi: 10.1021/acsami.3c15833 (PMC10895581; doi:10.1021/acsami.3c15833)
Supplement: Supplementary file 1 — am3c15833_si_001.pdf [file am3c15833_si_001.pdf]

Supplementary information for:

Flexible Magnetocaloric Fiber Mats for Room- Temperature Energy Applications

Vahideh Bayzi Isfahani[1,2]*, Indrani Coondoo[2], Igor Bdikin[3,4], Konstantin Skokov[5], João Ricardo da Silva Gomes[1], Rosa Maria Ferreira Baptista[1], Clara Rodrigues Pereira[6], João Pedro Araújo[7], Michael Scott Belsley[1], Etelvina de Matos Gomes[1], João Horta Belo[7]* and Bernardo Gonçalves Almeida[1]*

[1]Centre of Physics of Minho and Porto Universities (CF-UM-UP), LAPMET, Physics Department, University of Minho, 4710-057 Braga, Portugal
[2]Department of Physics & CICECO – Aveiro Institute of Materials, University of Aveiro, 3810-193 Aveiro, Portugal
[3]TEMA: Centre for Mechanical Technology and Automation, Department of Mechanical Engineering, University of Aveiro, 3810-193 Aveiro, Portugal
[4]LASI—Intelligent Systems Associate Laboratory, 4800-058 Guimarães, Portugal
[5]Institute of Materials Science, Technical University of Darmstadt, 64287 Darmstadt, Germany
[6]REQUIMTE/LAQV, Department of Chemistry and Biochemistry, Faculty of Sciences, University of Porto, 4169-007 Porto, Portugal
[7]Institute of Physics of Advanced Materials, Nanotechnology and Photonics (IFIMUP), Department of Physics and Astronomy, Faculty of Sciences, University of Porto, 4169-007 Porto, Portugal

** Corresponding Authors*

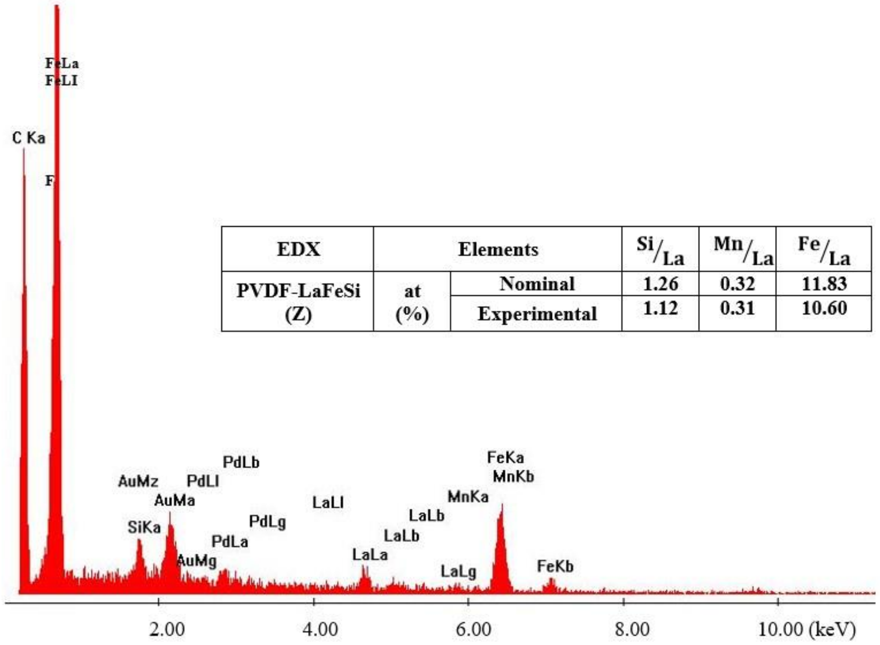

EDX	Elements		$^{Si}/_{La}$	$^{Mn}/_{La}$	$^{Fe}/_{La}$
PVDF-LaFeSi (Z)	at (%)	Nominal	1.26	0.32	11.83
		Experimental	1.12	0.31	10.60

Figure S1. EDS spectrum regarding the magnetic particle inclusion shown as Z in Figure 1 (d). The corresponding atomic percentages (atom%) of the particle elements are presentedd in the inset.

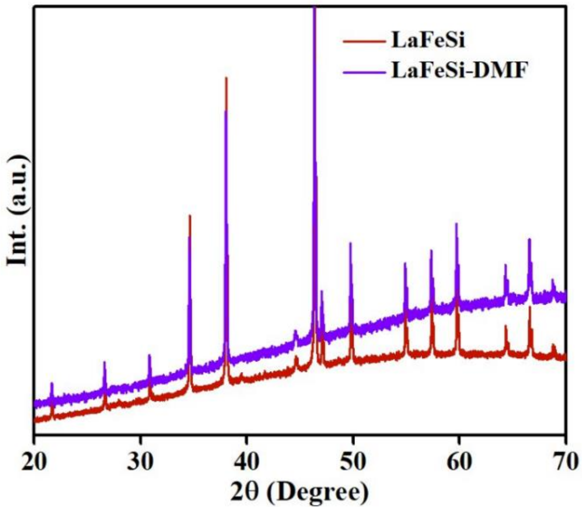

Figure S2. Comparison of the XRD spectra of the LaFeSi powder before and after it was dissolved in DMF.

DMF is a well-known solvent for the PVDF polymer. Therefore, to assess the stability of the LaFeSi powder after dispersion in DMF, a small amount of it was mixed with 2 cm^3 of DMF. Subsequently, the solvent mixture (DMF) was allowed to evaporate under room conditions, and the remaining powder was analyzed. Figure S2 displays the XRD spectra of the powder before and after dissolution in DMF. The similarity between both patterns suggests that LaFeSi remains stable in DMF, indicating its suitability for nanofiber preparation.

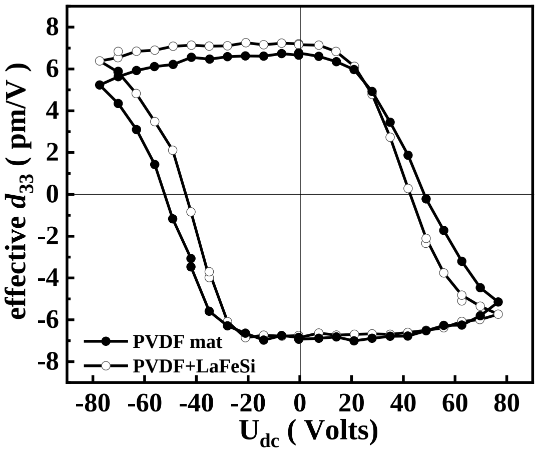

Figure S3. Comparison of the hysteresis loops of PVDF and PVDF-LaFeSi fibers obtained at ±75 V.
